# Implementing early childhood education for children with disabilities in South Africa and Kenya

**DOI:** 10.4102/ajod.v13i0.1326

**Published:** 2024-05-08

**Authors:** Brigitte J. Clark, Willene A. Holness, Ruth T. Nyamadzawo, Dennis Moogi

**Affiliations:** 1 School of Law, College of Law and Management Studies, University of KwaZulu-Natal, Pietermaritzburg, South Africa; 2 School of Law, Faculty of Social Sciences, Oxford Brookes University, Oxford, United Kingdom; 3 School of Law, College of Law and Management Studies, University of KwaZulu-Natal, Durban, South Africa; 4 Navi Pillay Research Group, Durban, South Africa; 5 Action for Children with Disabilities, Nairobi, Kenya

**Keywords:** early childhood education, children with disabilities, children’s rights, legislation and policy

## Abstract

**Background:**

The immediate implementation of early childhood education (ECE) for children with disabilities in South Africa and Kenya has been impeded by obstacles. Major gaps in implementation remain. We investigate, firstly, the widely held, but in our view fallacious, belief that the implementation of inclusive ECE can be progressively realised only when there are available resources. Secondly, we examine the other fallacious belief that children with severe and profound intellectual disabilities are ineducable, and thirdly, the belief that the provision of inclusive ECE is merely a regulatory governmental function, implying that accessibility and reasonable accommodation requirements for children with disabilities do not rest primarily on the state.

**Objectives:**

This study aimed to investigate the gaps in both countries between the policies and legislation and effective implementation, to show that these gaps are exacerbated by the perpetuation of these fallacious beliefs and by information vacuums at governmental level.

**Method:**

A critical analysis of inclusive ECE was undertaken on relevant law and policy processes in both countries to expose both governments’ reasons for their lack of effective implementation of inclusive ECE.

**Results:**

The factors contributing to the lack of immediate and significant implementation of inclusive ECE for children with disabilities in both countries have been investigated.

**Conclusion:**

Accountability and transparency need to be implemented at the governance level to ensure that both governments fully implement and prioritise inclusive ECE.

**Contribution:**

This article establishes that mistaken premises and information vacuums may be used by governments in an attempt to renege on their international and constitutional obligations to implement inclusive ECE.

## Introduction

From the perspective of human rights lawyers, academics, and experts in early childhood education (ECE) and child disability law, we investigate some widespread (but, in our view, fallacious) premises and beliefs about ECE, also known as early childhood development (ECD), for children with disabilities in Kenya and South Africa (SA) to expose the misconceptions about state responsibilities in this regard. We undertook a critical analysis and evaluation of relevant law and policy processes in both countries on inclusive ECE and identified a policy-implementation gap exacerbated by certain ideological notions and information vacuums. Both countries have undergone recent law and policy reform processes (Nthenge 2017; Philpott 2014), in particular, the provision of ECE is now the responsibility of the Departments of Education and not Social Development in both countries. This move occurred in 2017 in Kenya and in 2021 in SA. However, in both countries, the major obstacles preventing children from accessing quality education at ECE level include negative attitudes towards disability, and a lack of resources, funding, facilities and trained personnel to effectively cater to the diverse needs of children with disabilities (Dombrowski, Sitabkhan & Kilonzo 2023). Unfortunately, major gaps remain and the checking and evaluating of ECE programmes has been inconsistent and opaque at times, with scant regard for improving access to quality-inclusive ECE and no acknowledgement that the barriers to access are not insurmountable (Karisa et al. 2022; Samuels et al. 2015).

Children with disabilities require special attention and care in their ECE phase (WHO 2018). The early years of any child’s life are very significant in their development, and children with disabilities urgently require support from birth to fulfil their potential (Ashley-Cooper, Van Niekerk & Atmore 2019). The emphasis should be on the urgent need for states, firstly, to offer a comprehensive package of ECE, secondly, to improve disaggregated data collection, and thirdly, to allocate adequate funding for inclusive ECE (Almasri et al. 2023). While international law does not explicitly refer to a right to ECE, scholars have convincingly argued that such a right can be read into the right to access basic education (Fredman et al. 2022). International law obliges state parties to offer ‘affordable, accessible, quality, inclusive ECCE, with adequate resources’ (Fredman et al. 2022:1). The Convention on the Rights of the Child (CRC), the International Covenant on Economic, Social and Cultural Rights (ICESCR), and the Convention on the Rights of Persons with Disabilities (CRPD) consistently demonstrate that states must provide this inclusive ECE (Fredman et al. 2022). International law also requires states to take measures to ensure equality and inclusion of children with disabilities in the services provided to them, with reasonable accommodation where needed (CRPD art 2(4), art 24 (2) (c), CRC 28(1), ACRWC art 13).

This article does not aim to provide an exhaustive list of the applicable international law or legislative provisions as this has already been covered by other authors (Bekink 2022; Nthenge 2017). Instead, we focus on the need for critical analysis and evaluation of the existing laws and policies on inclusive ECE in SA and Kenya. We seek to investigate some of the beliefs or myths, which may impede the intended outcomes and delivery of equitable access. In this regard, Hayes and Bulat (2017) also dispelled various myths in relation to inclusive education in their seminal article on the negative impact of inclusive education on children without disabilities living in low- and middle-income countries such as SA and Kenya and many others globally. Those authors addressed the fallacious notions that inclusive education was more expensive than education in specialised (segregated) settings; that segregated options were more effective; that limited resources should translate into prioritising education of non-disabled students; and that education of children with disabilities was a luxury not available to low- and medium-income countries.

Firstly, we address the notion that inclusive ECE is not urgent and can be progressively realised when there are available resources. Secondly, we address the notion that children with severe and profound intellectual disabilities are ‘ineducable’. The last notion that we address relates to the assumption that inclusive ECE is only a regulatory duty for national governments, enabling them to bypass the accessibility and reasonable accommodation obligations resting on the state in this regard. A contextual background on Kenya and SA is provided, illustrating, not only the incoherent and lackadaisical implementation of policy and legislation (largely as a result of many of these premises listed above) but also the advances made from litigious intervention – particularly in SA.

## Contextual background

### Kenya

Since 1963, ECE has been an essential component of the Kenyan education system (Wanjohi 2011). International treaties have been directly imported and domesticated through article 2(6)13 of the 2010 Constitution, including the CRC and CRPD. Despite this, major challenges to accessing quality inclusive ECE involve cultural prejudice and negative attitudes, poverty, insufficient data, and inadequate tools for assessing and identifying children with disabilities still exist, (Sessional Paper No. 1 of 2019). Equitable access is further constrained by regional differences, a lack of inter-sectoral coordination (Neuman & Devercelli 2012:27), insufficient pre-primary centres, and a lack of adequate play, teaching and learning materials (Muthoni 2016). Parents of children with severe and multiple disabilities are frequently more vulnerable to poverty and cannot afford assistive devices required for these children (Odongo 2018:26). Although enrolment in ECE centres increased by 7% from 2015 to 2018, an annual decline of 19% followed in 2019 (Masinde et al. 2022).

The Sector Policy requires that the Ministry of Education develop and implement early identification, assessment and intervention standard procedures and guidelines for children with disabilities. The Education Assessment and Resource Centres (EARCs) were operationalised in 1984 to provide early screening and intervention services for children with disabilities. However, they are often unable to implement functional assessments. A lack of functional assessment expertise and structure affects the performance of multidisciplinary teams tasked with the assessment of children with disabilities (Emmy 2020). In some parts of the country, the Ministry of Health and the local county government do not formally work together in respect of EARCs, for instance, only 15% of EARCs utilise nutritionists and speech therapists (KISE 2018). School admission policies do not require disability screening or assessments to be undertaken (Muga 2003:33). Integrated data management systems for the early identification of appropriate assessment, identification processes and the placement of children with disabilities in ECE is lacking (Abubakar et al. 2022).

The responsibility for pre-primary education was devolved to county governments after the adoption of the 2010 Constitution. Thus, the oversight and management of pre-primary education is the responsibility of the county government (Sections 18(a) and 18(k) *Basic Education Act 2013*). County governments are responsible for independently budgeting and implementing ECE (Piper, Merseth & Ngaruiya 2018) and providing annual reports on educational progress to parliament (Nthenge 2017). As a result, the provision of ECE resources has been administered in the context of limited local funds for social sectors (Koech 2003).

Kenya’s development blueprint of 2008, the Kenya Vision 2030, recognises the need to address the barriers that children with disabilities and their families face when accessing education. The Kenya Vision emphasises the need to ensure that children with disabilities have equal opportunities to access quality education, and that their ‘special needs’ are considered in the design and implementation of educational policies and programmes (Sessional Paper No. 10 of 2012). The 2010 Constitution recognises the rights of children to compulsory and free basic education (art 53(1)(b)) and healthcare, and prohibits discrimination on the grounds of disability (article 54). Article 54(b) grants persons with disabilities the right to access educational institutions and facilities, which are ‘integrated into society and are compatible with the interests of the person’. The *Persons with Disabilities Act of 2003* provides for the protection and promotion of the rights of persons with disabilities, including children, and guarantees the right to education for persons with disabilities on an equal basis with others (art 18). It emphasises the need for educational institutions to accommodate the needs of children with disabilities, providing them with necessary support services to ensure their equal access to education, and sets out the general legal framework and principles for promoting inclusive ECE. The Sector Policy (Ministry of Education Kenya 2018) further commits the government to full participation in ECE, promoting inclusive education and advocating the right of children with disabilities to be enrolled in regular classrooms with their peers without disabilities. This shift to inclusive education also recognises the role of special learning institutions, special units in normal learning institutions and education, which is based at home to provide for children with severe disabilities. Thus, Kenya emphasises the need to specifically maintain special schools but is also trying to move towards inclusive education and a home setting where appropriate. This is a national policy where the local governments should align their ECE programmes with these policy statements.

The *Basic Education Act 14 of 2013* (the Act) regulates the provision of general education throughout the country. Aseka and Kanter (2014) expressed concerns about the Act’s focus on segregated education instead of inclusive education. The Act was the first education law to explicitly refer to ECE, including access for children with disabilities (Nthenge 2017). The Act provides for the establishment of special schools to provide special needs education at pre-primary, primary, and secondary school level (art 28).

The *Children’s Act of 2022*, replacing the 2001 Act, recognises and safeguards every child’s right to education and guarantees free compulsory basic education. This Act states that children with disabilities have the right to be treated with dignity and have access to appropriate free medical treatment, care, education and training (art 20(1)). The *Early Childhood Education Act* (2021) establishes a framework for systems to administer ECE in a county. This Act contains specific provisions that obligate the county governments to identify and undertake an assessment of children with disabilities and to ensure that these children are not discriminated against or prevented from accessing and completing their attendance in ECE. This national legislation now obligates each county to enact county-specific legislation and develop guidelines that will inform implementation of ECE programmes.

Recently, legislators have been championing for a disability-specific law to be enacted to advance the rights of children with disabilities in education. The proposed Learners with Disabilities Bill 2023 has been introduced in parliament and *explicitly* mandates the county governments to facilitate the identification and assessment of children with disabilities and creates a registry at the county level to provide for the screening and assessment of each child who may have a disability, even if they are not attending school (Part IV).

### South Africa

The history of ECE in SA dates back to the 19th century. However, under the apartheid government from 1948 to 1994 there was very little investment in ECE, especially for black children (Atmore 2013). Restrictions on welfare subsidies for black children were imposed as a result of limits on parental income, while well-funded government preschools were available only in the white education system (Nel 2007). Thus, most black children had limited access to education and often relied on paid childcare centres within the community catering for children aged 3–6 years (Rudolph, Millei & Alasuutari 2019).

In 1994, the democratic government recognised, through the Reconstruction and Development Programme (RDP), that children’s early experiences and learning have a significant impact on their future academic and personal success. This programme highlighted the need for comprehensive ECE services, including health, nutrition, education, and social services. Since then, several policies and initiatives have been introduced to promote ECE. Nutrition for preschool children apparently remains the responsibility of the Department of Health (Richter et al. 2019). Adequate nutrition for children with disabilities will require ensuring that children who fall through the cracks of the Department of Health’s surveillance are caught in the protective net of ECE services under the responsibility of the Department of Education. The Integrated Nutrition Programme (INP) and the School Health Programme (SHP) support ECE services. The INP was established in 1994 to tackle malnutrition and it incorporates various feeding initiatives such as the Primary School Nutrition Programme (PSNP), community programmes, and food parcels. As a multisectoral endeavour, the INP involves the Departments of Health, Social Development, and Agriculture. During the coronavirus disease 2019 (COVID-19) pandemic, the interruption of the INP had dire consequences for children’s health. However, litigation forced the Department of Education to roll out the INP without delay to school-going children (*Equal Education and Others v Minister of Basic Education and Others* 2021 (1) SA 198 (GP)). The SHP 2003 was implemented through collaboration between the Departments of Basic Education and Health with the support of other stakeholders and aims to support the holistic development of young children and to promote their health, well-being, and early learning. It is an integral component of the broader healthcare system, ensuring that children receive timely and appropriate health interventions that contribute to their holistic development. Through collaboration with schools, parents, and healthcare professionals, this service strives to create a supportive and nurturing environment that fosters the well-being of the younger generation, setting the foundation for a healthier and more prosperous future (Shung-King 2013:89). The programme identifies and addresses health issues, places a strong emphasis on education, prevention, and the creation of a supportive and nurturing school environment by ensuring the following:

- a health assessment, whereby children are screened for a number of health conditions such as vision and hearing
- health education and health promotion, age-appropriately tailored
- psychosocial and mental health assessments
- the identification and support of children with chronic health conditions
- facilitating the creation of safe and healthy school environments
- preventive interventions, mainly immunisations and deworming (the provision of sexual and reproductive preventive services at school is still under contestation)
- addressing minor ailments (Shung-King 2013:89).

The Constitution, 1996, recognises the rights of all children to access basic education, healthcare and social protection, and prohibits discrimination on the grounds of disability (Sections 27, 29, 28 and 9). The *Children’s Act 38 of 2005* provides for the protection and promotion of the rights of children, including those with disabilities, and prohibits unfair discrimination on the basis of disability (Sections 6(2)(d) and 11(b)). Furthermore, the Act requires that all children have access to ECE services and provides for the regulation of established ECE accessible to children with disabilities (Sections 91–103). The legislation appears to envisage an ECE system with ‘programmes’ being provided and funded through national and provincial strategies and with delegation to municipalities where relevant (Sections 92–94). However, the legislation does not refer to ‘inclusive’ education. In relation to inclusive ECE, the legislation specifies that ECD programmes should be ‘appropriate to the needs of the children to whom the programme is provided, including children with a disability, chronic illness and other special needs’ (Section 94(3) of the *Children’s Act*). Such wide and vague legislative instruction misses the target of inclusive education mandated by international law (Fredman et al. 2022).

The Education White Paper 5 on Early Childhood Development (WPECD) (Department of Education, Republic of South Africa 2001) highlighted the link between ECD programmes and child well-being, school achievement, and cognitive and other developmental domains. However, while the policy addressed the inequity in the provision of ECD programmes and the fragmentary ECD legislative and policy framework, it has been criticised for not fully delivering on these commitments (Storbeck & Moodley 2011). The link between ECD services and child development was not reflected by meaningful change in how ECD services were provided (Storbeck & Moodley 2011).

Guidelines for educators to identify and support children who may have barriers to learning and development were provided to educators and emphasised the importance of early identification and intervention to prevent long-term negative impacts on children’s education (Department of Basic Education *Policy on Screening, Identification, Assessment and Support 2014* [Department of Basic Education 2014]). A comprehensive process of screening, identification, assessment and support is outlined, including the use of standardised tools and collaboration with parents and other professionals. However, apparently, many ECD centres are either not aware of the need to conduct assessments for disability or lack the necessary resources to do so (Karisa et al. 2022). This finding is based on the results of an audit of ECD centres in 2014, which showed that assessments of children with disabilities are generally very low across all disability types (Department of Education’s *National audit of ECD services* [Department of Social Development 2014]).

The National ECD Provisioning Audit 2000 conducted by the Department of Basic Education (DBE) to ascertain the status of ECD in the country, unsurprisingly had found that access to ECD was generally low and that in the 5–6 year age group, only 43% of children were accessing ECD. Tellingly, the audit stated:

While the findings of the Audit established how few disabled learners are currently enrolled in ECD sites, little is known about these disabled learners. Research focusing more specifically on learners with disabilities would allow for more responsive and appropriate provisioning. (Department of Education 2001:168)

Almost 15 years later, another audit by the Department of Social Development (DSD) found that ECD centres generally tend to screen and assess the disabilities of children with particular disabilities such as ‘behavioural challenges’, developmental delays and ‘learning disabilities’. The authors of the 2014 audit posited that the fact that the number of children with such types of disabilities was higher than the number of children with other disabilities was largely because ECD centres were more knowledgeable about how to conduct assessments for these disabilities. More generally, the number of children with disabilities of various types obtained from the audit may not be reflective of the actual number of children with such disabilities because of the national lack of disability assessments carried out by ECD centres (DSD 2014:111). The audit found that it is likely that ‘there are children with undiagnosed disabilities, which implies that these children are not receiving the care they need’ (p. 113). The audit also noticed that because assessment of disabilities can be accessed independently from ECD centres, by implication the curriculum and care may ‘not [have] been tailored to meet the special needs of some children’ (p. 113). The DSD recommended training and awareness on disabilities or developmental delay to teachers and caregivers to enable early identification. Concerningly, while the audit collected data on accessibility of infrastructure, it did not obtain data on potentially exclusionary admission requirements or on whether reasonable accommodations were offered to children with disabilities. The accessibility of ECD centres for children with disabilities paints a bleak picture, with suitable physically accessible toilets found at less than 50% of registered centres and only 17% of ECD centres with wheelchair ramps, and only 9% with handrails. Some 63% of registered ECD centres reported that their classrooms were accessible to children with disabilities. However, a major flaw of the data was that special modifications were unlikely to be made to accommodate children with disabilities, presumably based on the ECD practitioners’ belief that their classrooms *were* accessible as there were no specific requirements for rating the classrooms’ accessibility (DSD 2014:222).

In 2015, the National Integrated Early Childhood Development Policy (NIECD Policy) aimed to promote comprehensive inclusive ECE services. While the policy recognised the significance of early intervention and established screening and assessment programmes to identify children with disabilities, a lack of resources and support hindered the development of individualised education plans for children with disabilities (Clark, Naidoo & Lilenstein 2019:2, 9). From 1996, the Interim Department of Education Policy for ECD recognised the complexity of ECE and established a national ECD pilot project for implementing a universal reception year. It defined ECD as a comprehensive concept that encompasses the physical, mental, emotional, spiritual, moral and social growth and development of children from birth to at least 9 years of age. Sadly, there is a paucity of information on the benefits of ECD interventions for children with disabilities and on the attendant costs relating to accessibility and appropriateness in the NIECD (Desmond et al. 2019:282). Desmond et al. (2019) stressed the need to develop more effective and cost-effective models, such as non-centre-based approaches, and including home-visiting and playgroups.

In 2020, the Children’s Amendment Bill ([B 18B 2020] No. 43656) aimed to provide ECE services for children with disabilities who were above school-going age until the year before these children entered school, and to provide an inclusive ECE system. However, this Bill was unsuccessful in dealing with the consequences of the transition of ECE from the Department of Social Development to Basic Education. Those effects are staggering, with registration woes affecting the provision of services to these children (Ally, Parker & Peacock 2021). The registration process for early learning centres in South Africa is both time-consuming and expensive. According to the 2021 ECD census, a substantial 60% of these centres operate without official registration. This situation often arises not by choice but because of the intricate challenges involved in meeting the demanding norms and standards, which vary across municipalities and are further complicated by local by-laws. To attain compliance, early learning facilities must navigate a complex set of requirements, including obtaining land use and zoning certificates, securing a fire clearance certificate, undergoing environmental health inspections, and completing exhaustive application forms (Innovation Edge 2023). Many schools have made repeated efforts to meet these criteria but find themselves consistently falling short. As a consequence, a considerable portion of their resources, already limited, is allocated to the registration process, presenting a significant setback for these centres and others in South Africa facing financial constraints. This challenge underscores the urgent need for a more streamlined and accessible registration framework, considering the financial constraints faced by many early learning centres in the country (Innovation Edge 2023). The Bill also failed to deal with the issues faced by unregistered and unsubsidised ECD centres lacking in resources. The Bill’s proposal to change the peremptory nature of funding for poverty-stricken communities and children with disabilities from mandatory to discretionary serves to exclude these children from ECE. Venter (2022) identifies the devastating impact of family or community poverty on children in the ECE phase. The mitigation of poverty is a cross-cutting obligation on many state departments and the provincial and local government budgeting for these groups of children should be an urgent priority. The *Children’s Amendment Act of 2022* commenced in January 2023. However, the Bill’s draft provisions on ECE were abandoned during 2021.

The SA Law Reform Commission (SALRC) seeks to domesticate the CRPD by either the enactment of a disability-specific Act or by the amendment of the *Promotion of Equality and Prohibition of Unfair Discrimination Act of 2000* (PEPUDA), which prohibits unfair discrimination against persons with disabilities (SALRC 2020). The current anti-discrimination provisions in PEPUDA have been successfully used by children with physical disabilities (seeking reasonable accommodations and accessibility in private schools and shops open to members of the public) (*Oortman v St Thomas Aquinas Private School* Case 1/2010 Witbank Equality Court (unreported) (Holness & Rule 2014); *Haskin v Khan* (EqC) unreported case number 03/19 Mitchell’s Plain 2020, discussed in Holness (2022). Surprisingly, however, the Equality Courts have not yet received a complaint of discriminatory admission, a lack of accessibility, and a lack of reasonable accommodations by a parent or a child with a disability in the ECE phase. It seems that the domestication process is not prioritising legislative protection for inclusive ECE. Furthermore, there is widespread misunderstanding and underappreciation of the need to offer ECE to children, not just in the reception years, but from infancy: policy and law reform needs to prioritise children from birth (Richter et al. 2019). The White Paper on the Rights of Persons with Disabilities (WPRPD) highlights six areas that need attention to assist persons with disabilities, with a focus on ECD. It recommends that children with disabilities must have access to ECD programmes and facilities. The WPRPD further highlights the importance of addressing ECD as a foundational element in the broader strategy to empower and support persons with disabilities, ensuring that they have equal opportunities for growth, learning, and development from the very beginning of their lives (Department of Social Development 2016).

## Are the South African and Kenyan governments only mandated to implement inclusive early childhood education progressively and depending on the availability of resources?

Inclusive education is often misunderstood as very costly and impractical, but inclusive models of education are less expensive than segregated ones (UN-DESA, OHCHR & IPU 2007). Inclusive school settings may in fact reduce expenses in comparison to special schools (Halvorsen 1996 cited in Mezzanotte 2022; Odom, Parrish & Hikido 2001). The broad scope of inclusive education requires a comprehensive and intersectoral commitment that cuts across government (General Comment 4 on Article 24 of the CRPD (2016 para 61)). Both SA and Kenya are committed to intersectoral collaboration on ECE (Kenya’s Sector Policy 2018 para 4.12 and SA’s ECD paras 7.2, 7.3.3.5). However, the implementation is frequently hampered by overlapping mandates, buck-passing, and a failure to prioritise budgets (Hudson, Hunter & Peckham 2019:6).

There is a widely held premise that these policies on ECE, particularly for children with disabilities can only be gradually implemented, depending on the reasonable availability of adequate funding. Ostensibly, both governments rely on this premise to justify non-compliance with state obligations and as an excuse not to act expeditiously. On the contrary, all children *urgently* need and should have an *immediate* right to inclusive ECE (Vargas-Baron et al. 2019). Neuroscience has established the nature of early brain development and the need to support parents and caregivers to ensure that all children fulfil their potential (Black et al. 2017). Arguments based on the premise that only progressive realisation of this right is required should not allow governments to delay or renege on the obligations of state parties to move towards expeditious implementation of inclusive ECE (Chenwi 2013:744). In Kenya, immediate equitable budgetary allocation is needed with continual review by the Minister of Education in the Sector Policy (Ministry of Education Kenya 2018:30). In the 2022–2023 budget, the Government allocated Ksh. 544.05 billion to education – 16.36% of the national budget and 4.30% of GDP (Human Rights-Based Analysis of Kenya’s Budget 2022/23, OHCHR, Nairobi). Although the national budget allocation to the education sector is within international agreed benchmarks or slightly below the Dakar Commitment on Education for All by the African Union, it is difficult to clearly determine what is budgeted for ECE. This is because at the national level, the ECE budget is merged with that of primary education. The responsibility to deliver quality ECE is devolved to the counties. The national government allocates resources for pre-primary programmes with the counties deciding how to spend the funds. This results in variation in the standards and implementation of ECE policy across the different counties and general underfunding. Furthermore, capital expenditure items including physical infrastructure are prioritised and the expenditure involved in training of staff and quality assurance is overlooked.

The immediacy of the right to education is recognised in the SA Constitution (Section 29(1)(a)), which provides that everyone has the right to a basic education. The Constitutional Court confirmed that the right to basic education is not subject to *progressive realisation* and instead is *immediately* realisable without internal limitation (*Governing Body of the Juma Musjid Primary School & Others v Essay N.O. and Others* 2011 (7) BCLR 651 (CC)). Such a limitation on other socio-economic rights usually merely requires that those rights are progressively realised within available resources or subject to reasonable legislative measures. The right to a basic education in Section 29(1)(a) may only be limited by a general law that is ‘reasonable and justifiable in an open and democratic society based on human dignity, equality and freedom’ (Section 36 of the Constitution). Furthermore, the Constitutional Court has adopted a contextual method of interpretation in this regard by understanding rights in their social and historical context (*Government of the Republic of SA v Grootboom* 2001 1 SA 46 (CC) para 25). A contextual interpretation of the right to basic education should prioritise the provision of free basic education to disadvantaged children, in particular inclusive ECE (Arendse 2011; Fredman et al. 2022). Both governments are thus obliged to make education *immediately* available, accessible, acceptable and adaptable for *all* children (the 4-A scheme) (see Committee of the CRPD’s Concluding Observations on various countries, as cited in Fredman et al. 2022).

The constitutional right to basic education creates a positive right that basic education be provided for every person, not merely a negative right that persons should not be prevented in pursuing their basic education (*Ex parte Gauteng Provincial Legislature: In re Dispute Concerning the Constitutionality of Certain Provisions of the Gauteng School Education Bill of 1995* (1996 (3) SA 165 (CC) para 9). Therefore, the state is obliged to take positive steps to ensure that basic education is provided. Thus, the right to basic education is not merely a right of access and is not subject to internal qualifiers (see Section 29(1)(b)). Section 29(1)(a) differs from the right to *further* education, which is ‘qualified’ to the extent that ‘[t]he state must take reasonable legislative and other measures, within its available resources, to achieve the progressive realisation’ of this right. The right to ECE, falling under the right to basic education it is submitted, cannot be limited as a right to access or be subjected to internal qualifiers.

The civil society initiative, Real Reform for ECD, exposed some of the registration barriers and continues to seek solutions at local government level for better budgetary allocation to the sector (Mantjé 2022), but it excludes provision for children with disabilities. Despite the SA government’s intentions to provide quality ECE for all children in SA, articulated in its National Intergrated Early Childhood Development (NIECD) Policy, the realisation of this goal has been limited and educational reform has stagnated with little will to prioritise ECE for children with disabilities, as indicated in the SA Children’s Amendment Bill (Holness et al. 2023).

In the Kenyan case of *John Kabui Mwai & 3 others v Kenya National Examination Council & 2 others* [2011] eKLR. 220J, the court recognised that realising socio-economic rights entails achieving improved conditions for the poor and less advantaged members of society. However, the government failed to provide evidence of concrete policy measures, genuine commitment, guidelines, and tangible progress towards achieving the right to education, and progressive realisation is not an indefinite defence (*Michael Mutinda Mutemi v Permanent Secretary, Ministry of Education & Ors* [2013] eKLR, Petition No. 133 of 2013).

Neither SA nor Kenya prioritise ECE sufficiently (Neuman & Powers 2021), and children with disabilities are even less of a priority (Almasri et al. 2023). Countries with compulsory and free ECE, such as Peru and Ghana, have greatly increased children’s access to ECE for children with disabilities (Neuman & Powers 2021). Despite international pressure on states to prioritise ECE, some countries reflect ‘a lack of sustained follow-through, resource provision’ and a general failure to turn advocacy into donor or government financed investments (Neuman & Powers 2021). Choices in governance of ECE systems would ensure quality in service-provision, affordability, promotion of cost-effectiveness of the preferred interventions, and achievement of equity and access (Kagan & Cohen 2005). Effective governance can promote coherence in policymaking and meet the diverse needs of children and families across disparate geographical areas and socio-economic statuses.

## Are children with severe and profound disabilities ‘ineducable’?

Some children with disabilities, particularly those with more severe disabilities such as developmental (e.g. autism) or intellectual disabilities, in ECE and later in compulsory schooling ages, have been labelled as ‘ineducable’ (Special Rapporteur on the rights of persons with disabilities 2017). The Special Rapporteur has acknowledged the prevailing myth of ineducability. The Special Rapporteur has stressed that the state obligation to ensure that support is provided to persons with disabilities in the educational context is:

[*N*]ot a single transversal obligation established in the [*CRPD*], but also a requirement that derives from the basic principles of human rights, such as dignity, universality, individual autonomy, equality and non-discrimination, participation and inclusion. (p. 1)

Despite these comments of the Special Rapporteur, the South African state has sought to argue that children with severe and profound intellectual disabilities should not qualify for admission to special schools, that no amount of education benefit them or be of value to them (*Western Cape Forum for Intellectual Disability v Government of the Republic of SA and Another* (2011 (5) SA 87 (WCC) Paras 3.9 and 17). In this case, the court found that the state has a duty to provide equally for the education of all children which include children with severe and profound disabilities. The state had tried to rely on the progressive implementation of its policy as a defence to immediate provision of education to children with severe and profound intellectual disabilities (Paras 3.9 and 17). This defence would have served to totally exclude from accessing educational facilities for many generations (Ngwena & Pretorius 2012). The High Court found that the only education available in the Western Cape province for such children was at Special Care Centres run by NGOs (para 3.4). The court laudably (Ngwena & Pretorius 2012) admonished the state for declaring that these children were ‘ineducable’ and held that the provision by the state for such children was significantly less than that provided for other children; was inadequate for their educational needs; and was made available *only* where an NGO and not the government provided such facilities. This lack of governmental provision for inclusive ECE violates ‘the rights of these children to education, equality, human dignity and protection from neglect and degradation’ (para 4 of the judgment). Relying extensively on international and constitutional law, the court concluded that the state had breached these children’s rights to a basic education (paras 6–27) and their constitutional right to equality (paras 27–44). The court ordered that the staff of such Special Care Centres should receive ‘proper accreditation, training and remuneration’ and the state was ordered to show what steps they had taken to give effect to this order (para 45). Delays in enforcement of the order have, however, been noticed (Wood et al. 2018). Accordingly, government implementation of the court order appears to pay lip service to the identification of such breaches and the need to act, with a sense of urgency, to remedy them. Blanket bans or exclusions therefore cannot be countenanced, particularly in the ECE sector, and are based on fallacious and misguided beliefs.

In the Kenyan context, the belief exists that children with ‘severe’ disabilities are ‘ineducable or that their management in regular education settings strains the scarce financial resources’ (Chomba et al. 2014). Families and educational institutions argue that providing education and support to children with severe disabilities requires significant resources (WHO 2012:25). Community stigma against children with disabilities may mean they suffer exclusion, ostracisation and are sometimes ‘locked’ up or hidden (Hirpa DA 2021:5). This persistent stigmatisation also affects the access to ECE and food supplementation programmes (Zuurmond et al. 2016). Notably, there is no explicit ‘zero reject’ requirement in Kenyan law regarding inclusion, which means that schools have the discretion to refuse admission to a child deemed ineducable, so potentially discouraging parents from seeking assessments and other special education services, which then disadvantages the children (Odongo 2018).

## Do states only have a ‘regulatory’ duty in relation to early childhood education?

Governmental accountability is frequently scuppered by its stance that its duty is merely to regulate the provision of ECE provided by private stakeholders, usually in ECE centres, and excluding other types of ECE provision such as home-based care (Clark & Holness 2022). The reliance on this false notion of accountability sidesteps the fact that the rights to health and education are cross-cutting obligations for states, in particular where families cannot provide for their children, to which the pervasive poverty and inequality gaps in both Kenya and SA attest. The Diagnostic ECD Review (Richter et al. 2012) found that important gaps existed in the area of:

[*S*]upport for parenting, prevention of stunting among young children, safe and affordable child care for very young children and other families needing assistance, and planned rapid expansion early child care and education and provision of services to the most at-need families, including children with disabilities. (p. 1)

The SA NIECD policy has been welcomed for its emphasis ‘on interventions and support during pregnancy and the first two years’ (Röhrs et al. 2016:14). The policy adopts a phased-in approach to implementation, which will need financial investment (Desmond, Richter & Martin 2016). Röhrs et al. (2016:14) observed that the policy does not give maximum support to the invaluable role of NGOs in ECE through knowledge and expertise, resources and service delivery. This policy continues to promote the notion that ECE is ‘about centre-based policy … for children 3–5 years of age despite the comprehensive nature of the integrated policy (Shung-King et al. 2019:72). Rudolph et al. (2019) notice that the state’s technocratic approach to using data practices when prioritising solutions for ECE does not facilitate the required social change.

In order to establish a functional system for delivering ECE services, proper allocation of resources through sufficient provision of financing, human resources, infrastructure, materials, and support services is necessary (Section 98 of the *Children’s Act*, Berry, Dawes & Biersteker 2013). The availability of subsidies for ECE centres is contingent upon their registration, and yet becoming registered requires meeting minimum requirements outlined in the *Children’s Act* (Richter et al. 2012; Thorogood et al. 2020). It has been argued that these requirements, along with the necessary norms and standards, pose significant challenges for many centres, in particular those in impoverished and rural communities (Thorogood et al. 2020). By imposing stringent registration requirements, the government inadvertently exacerbates the inequities in ECE service delivery, as the lack of registration hampers access to vital subsidies, thus impeding provision of quality ECE. Expanding the scope and coverage of subsidies for ECE is essential to broaden and enhance accessibility (Wills & Kika-Mistry 2021).

Early childhood development conditional grants were implemented in 2001–2002 to extend ECE services. The conditional grant is for infrastructure and maintenance and supports additional funding for ECE subsidies (Wills & Kika-Mistry 2021). Conditional grants aim to assist ECD centres not fully funded from equitable share, conditionally registered centres and non-centre-based ECE programmes. The allocation is R17.00 per child for 264 days, while for non-centre programmes it is R6.00 per child per session (Parliamentary Monitoring Group 2022). In 2022, a ‘real-term decline’ of medium-term funding allocation for ECE was identified because, despite the increase in ECE conditional grants, the below inflationary impact translated into a decrease of 2.8% by 2024–2025 (Section27 2022). This decrease over time means that indigent children will be especially affected by this and by the proposed spending on infrastructure for low-cost ECD centres and maintenance of existing centres (Section27 2022). In 2023, the ECE grant allocation was increased, but ECE access for 1.3 million children aged 3–5 years who are currently excluded from ECE was not necessarily secured (Metelercamp 2023). This grant increase might not have been substantial enough to ensure enrolment for all children in this age group (Metelercamp 2023). In addition, the grant for Learners with Profound Intellectual Disabilities was reduced and under-spent (Metelercamp 2023). This raises questions about the transparency and clarity in the allocation process. On the one hand, there is an absence of data in the ECE grant increase. There is also a concerning reduction and under-spending of the grant for Learners with Profound Intellectual Disabilities. These issues underscore the need for clarity and thorough examination of both inclusionary aspects and the allocation and utilisation of specific grants, as indicated by the Parliamentary Budget Office in 2021, which highlighted that there was under-provisioning for learning and support materials (Parliamentary Budget Office, 2021).

Furthermore, the WPRPWD (2016:6.4.1.6) recognises that children with disabilities require a range of disability-specific support such as screening, early identification and assessment to determine individualised support programmes, language and communication development, assistive devices, and technology and therapy. The policy further mandates the development of a national integrated referral and tracking system with the Strategy and Framework for Disability and Rehabilitation (Philpott 2018). Kamga (2016) stated that, although the WPRPD is valuable, its implementation requires effective monitoring.

An example of policy and regulatory misunderstanding of the SA state obligation towards children with disabilities was found in the exclusion from resumption of schooling during the COVID-19 pandemic. The state’s irrational decision-making in relation to the provisioning for children with disabilities during the humanitarian crisis has been heavily criticised (Kamga 2021). The DBE was criticised for irrational delays and exclusions of some children with disabilities from returning to school after the hard lockdowns ended and it had to amend their regulations to ensure equitable return to school (*Centre for Child Law v Minister of Basic Education* High Court of Pretoria, case no 3123/2020 (unreported), Kamga 2021). The phased return to school regulations only provided for autistic, deaf, hard of hearing, blind and partially sighted children, but not for those with physical disabilities, intellectual disabilities, epilepsy and severe to profound intellectual disabilities. The court order indicates that the state cannot renege on its obligations on infrastructural and accessibility requirements, even at a time of a humanitarian crisis.

Two further SA cases brought during the pandemic illustrated governmental recalcitrance and perhaps an inadequate sense of urgency shown towards children in ECE. The first case, *Skole-Ondersteuning Sentrum NPC and Others v Minister of Social Development and Others* related to continued indefinite and blanket closure of ECD programmes once hard lockdown regulations were lifted (paras 14–17, 30). The Court declared that all private ECD centres were entitled to reopen immediately (para 51 read with para 1). The second case rested on the ability of ECD centres to remain open in the face of withheld or late payment of government subsidies to the centres in *SA Childcare (Pty) Ltd & Others v Minister of Social Development & Others*. In that case, the court ordered government to pay subsidies to qualifying ECD providers (Ally, Parker & Peacock 2022). Later, an appeal was less successful in *Minister of Social Development v SA Childcare (Pty) Ltd & Others* [2022] ZASCA 119 (29 August 2022) (unreported), where the court found insufficient evidence of continual breaches of constitutional obligations to the concerned ECD centres. These cases did not interpret the legislation or policies relating to ECE because the cases emanated from challenges to the *Disaster Management Act*. The state’s interpretation of its duty as merely regulatory was clearly evidenced in these cases.

It is hoped that the prioritisation of funding for infrastructure in the ECE sector, as per the current Children’s Act, will be more strongly enforced. The DBE has been forced through litigation to ensure that its regulations on norms and standards in schools are compliant with the Constitution (*Equal Education and Another v Minister of Basic Education and Others* 2019 (1) SA 421 (ECB para 44)). Similarly, infrastructural regulations to promote accessibility and inclusive ECE of children with disabilities will need to have stipulated timeframes for implementation, and require inter-governmental cooperation in relation to resource allocation and responsibilities. Ultimately, the SA state has effectively contracted out of providing ECE as a state service through its subsidisation policy. The ECE Stimulus package, rolled out during the pandemic when the closure of the sector created havoc for children and families across the nation, is an example of the state starting to take ECE provision seriously. However, that roll-out was not without problems (Gontsana 2021).

Local governments in SA and counties in Kenya are key implementers of policy and legislation, although county regulation of ECE in Kenya is mired in obstacles (Mantjé 2022). The implementation of minimum standards on universal access for children with disabilities at ECD centres operated by NGOs (which are not the same standards applicable to the public sector under the National Building Regulations) is both ‘wide-ranging and costly’ (Project Preparation Trust 2019:80). However, ECD facility design should provide for children with all disabilities, including behavioural, autistic, and intellectual disabilities requiring accommodations and accessibility to be promoted for their sensory integration (Project Preparation Trust 2019:80). Some ECD centres have been forced to allow the continued attendance of children up to 8 years old without an alternative educational option available to them (Project Preparation Trust 2019). Although the role of local government in relation to ECE is ostensibly limited to health and safety checks, Peacock (2023) argues that the absence of a coherent strategy and legislation on local government duties on ECE, including its building and upgrading of facilities, is legally suspect. Peacock proposes amendments to the *Municipal Systems Act* and *Children’s Act* to address the gap in explicitly allocated ECE local government powers.

## Conclusion

Although both governments have made some efforts to promote the rights of children with disabilities at the ECD level, wide disparities exist between legislation and policies and their implementation. Kenya’s resource and infrastructure barriers hinder the provision of quality inclusive ECE. However, perhaps the greatest difference between Kenya and SA is that the CRC and CRPD were directly imported and domesticated through article 2(6)13 of the Kenyan 2010 Constitution. By contrast, SA’s ratification of instruments such as the CRC and the CRPD still requires domestication (Sucker 2013). Thus, there may be more traction for accessibility and reasonable accommodation to be included in legislation and policy in Kenya, with a slower uptake in SA. As a result, Kenya displays a greater degree of political will than SA to implement inclusive ECE, although ECE centres in public primary schools continue to be unregistered with little or no data on their management (Ministry of Education Kenya 2018:1). Unfortunately, a continued lack of data on inclusive ECE translates into inadequate financing as the information vacuums are also relied on by the state to renege on its obligations. The under-prioritisation of early intervention, screening and assessment initiates the continued neglect of inclusive ECE into school level. Both governments have neglected adequate data capturing systems (Bekink 2022) and effective monitoring and evaluation (Griffin 2018). South Africa continues to neglect inclusive ECE law and policy reform. While Kenya’s reforms acknowledge the right to inclusive ECE, their inadequate implementation is at times aimed at political point-scoring without addressing persistent systemic challenges.

This article further illustrates that some of the premises relied on by the SA and Kenyan Governments, which have contributed to the slow implementation of government policy and legislation in this area. South Africa fails to expeditiously implement ECE legislation and policy and needs to learn from Kenya’s more urgent prioritisation of inclusive ECE. However, the lack of adequate budgeting, financing and monitoring of inclusive ECE in both countries is evidence of a breach of the states’ international law obligations for which they should be held accountable. While accountability mechanisms are in place for both governments, these are underutilised. The utilisation of reporting and complaint mechanisms to the treaty monitoring bodies may, in part, hold the states accountable. Still, these mechanisms have been stalled by the mistaken premises outlined in this article, which both states have used to argue their case when challenged on their poor provision and implementation of inclusive ECE.
